# Correlation of temperature-sensing microchip and rectal temperature measurements in cats

**DOI:** 10.3389/fvets.2023.1319722

**Published:** 2024-01-08

**Authors:** Marta Goig, Javier Godino, Maria Teresa Tejedor, Federica Burgio

**Affiliations:** ^1^GCTO MSD Spain, Madrid, Spain; ^2^MSD AH Animal Health Spain, Madrid, Spain; ^3^Department of Anatomy, Embryology and Animal Genetics, CiberCV, Universidad de Zaragoza, Zaragoza, Spain

**Keywords:** cats, microchips, monitoring, temperature, correlation

## Abstract

**Introduction:**

Rectal temperature (RT) is the reference standard for clinical evaluation of body temperature in mammals. However, the use of a rectal thermometer to measure temperature can cause stress and other problems, especially in cats. There is a need for clinical techniques that reduce both stress and defensive behavior as part of the provision of better medical care. Subcutaneous temperature-sensing identification microchips fulfil the current legal requirements and provide a reading of subcutaneous temperature (MT).

**Methods:**

The clinical study tried to determine whether there is agreement between MT and RT in normal (*n* = 58), hospitalized (*n* = 26) and sedated/anesthetized (*n* = 36) cats. Three measurements were taken using both methods (MT and RT) in each cat. Correlation between MT and RT, and differences between MT and RT, were estimated for pairs of data-points from the same individual, and all data pairs in each group were considered overall.

**Results:**

There was a strong positive correlation between MT and RT (*r* = 0.7 to 1.0) (*p* < 0.0005). The mean differences (*d*) were always negative and although statistically significant, these *d* values are likely of no biological importance. The overall *d* was ‑0.1°C in normal cats (*p* < 0.0005), -0.1°C in hospitalized cats (*p* = 0.001) and -0.1°C in sedated/anesthetized cats (*p* = 0.001). The limits of agreement between MT and RT appear narrow enough for MT to be acceptable estimate of RT. The overall limits of agreement (95%) were ‑0.71°C and 0.53°C (in normal cats); ‑0.51°C and 0.34°C (in hospitalized cats) and ‑0.60°C and 0.42°C (in sedated/anesthetized cats).

**Discussion:**

MT may provide a good alternative to RT measurement in cats. However, this study was mostly performed in animals that were normothermic. Therefore, further studies in larger groups of cats under different conditions are needed to compare trends and assess variation with time.

## Introduction

Body temperature differs both within and between individuals due to factors like age, sex, health status, physical activity, and stress levels (1–4). External factors, including ambient temperature, can also affect body temperature measurements. Temperature can be measured using different methodologies (e.g., rectal thermometer, auricular thermometer, axillary temperature measurement, infrared thermography, temperature-sensing microchip), which may yield different measurements. Rectal thermometry may be affected by several factors like digestion, peristalsis, muscle tone, and physical activity (5). Changes in tympanic membrane temperature can be explained by fluctuations in the hypothalamic temperature, while there is a lag between changes in rectal temperatures and shifts in hypothalamic and tympanic membrane temperatures (6). Environmental conditions are considered influential factors when temperature-sensing microchips (7) and axillary temperature (8) are used. Infrared thermography is also strongly influenced by environmental and biological factors, including the anatomical regions chosen for measuring temperature on the basis of blood flow (9–12). Understanding the factors that can influence body temperature is needed to interpret temperature readings and use these in assessing health status.

The core body temperature is the temperature of the internal organs of the body. The best sites for estimating core body temperature are the pulmonary artery, using a catheter, or the esophagus (13). Core body temperature is not measured in general practice and is usually estimated by means of rectal temperature measurement (RT) (14). In fact, RT is considered as the reference standard for clinical evaluation of core body temperature in animals, since there appears to be good agreement between these measurements (15). In dogs, agreement between core temperature and rectal temperature was 94.28–100.00% (16).

RT measurement is time consuming, stressful for animals and may lead to cross-contamination of gastrointestinal bacteria (17). Under some circumstances RT measurement can be very difficult, e.g., where individual temperature measurements need to be taken from a group of animals or under field conditions (7). It is also important to measure temperature correctly, e.g., when using a thermometer, to avoid inaccuracy or inconsistency. For example, the presence of feces in the rectum can interfere with the measurement of RT and presence of perianal or rectal diseases can make RT measurement difficult (16). RT measurement is very important for assessing feline health; however a wide range of reference intervals has been published for healthy cats (low end: 37.7–38.1°C, high end: 39.2–39.5°C) (18–20). This variation can be explained by the potential disparity of the conditions under which the measurements were taken; no details were given in the original references. However, Levy et al. (21) reported a range of RT in confined, healthy adult cats of between 36.7 and 38.9°C (lower than reported previously) when the ambient temperature was 20.3–30.8°C. According to these results, RT was not significantly correlated with ambient temperature. In cats, fever has been variably defined as 39.0–41.1°C (18–20).

Stress is considered as a factor influencing the recovery of veterinary patients. For this reason, clinical techniques reducing stress and defensive behavior are recommended, especially in cats (22). Stress-related defensive behavior might cause an increase in body temperature and heart and respiratory rates and/or can increase blood glucose concentrations (stress hyperglycemia), which could lead to erroneous diagnostic conclusions. When measurement of RT is required, then it might help if this is taken at the end of the physical examination, with the cat placed in a comfortable position, thus allowing the anus to be accessed easily without hyperextension of the tail. However, the use of less invasive methods of measuring temperature are recommended where possible. For more information, see the 2022 AAFP/ISFM Cat Friendly Veterinary Interaction Guidelines (23).

Previous studies in mice have shown the utility of temperature–sensing microchips (24, 25). In Spain, dogs, cats, and ferrets must now be identified by a microchip, under the Protection of Animal Rights and Welfare Act 7/2023 that came into force on March 28 (26). Thus, temperature-sensing microchips fulfill the legal provisions for both the identification of the individual and enable the measurement of body temperature at the subcutaneous implantation site (27). No retraining is needed to enable veterinary teams to assess temperature measurements taken by these microchips, resulting in easier and safer management of small animals in veterinary clinics.

The skin or body surface temperature is thought to be several degrees below RT (28). This difference ranged from −2.1°C to 3.6°C (8) in cats and from −1.3°C to 0.5°C in dogs (29). In children, the mean difference RT - surface temperature was only 0.29°C (30). In contrast, subcutaneous temperature measured using a microchip (MT) did not differ significantly from RT in the common marmoset (31), rat and mouse (32) and goat (33). Thus, we hypothesized that there would be a good correlation between MT and RT, measured using a digital thermometer, in cats under different conditions (normal, hospitalized, and sedated/anesthetized). Therefore, we assessed the correlation and differences between using paired data obtained from each cat.

## Materials and methods

### Animals

A total of 120 cats were analyzed in 11 clinics (A-K). A power calculation was not performed to determine the sample size. All available animals that met the inclusion and exclusion criteria (see below) were included in the study. The inclusion criteria for veterinary clinics to participate in this study were specialization in feline medicine and varied geographical distribution, representing the different regions of Spain, including the Balearic and Canary Islands. Data were collected from February 2022 to September 2022, while the new Protection of Animal Rights and Welfare Act 7/2023, which has made microchipping mandatory for dogs, cats, and ferrets, was being discussed by the Spanish parliament (26).

This study complied with the ARRIVE guidelines (34) and EU Directive 2010/63/EU on the protection of animals used for scientific purposes (35). The Ethical Advisory Committee for Animal Experimentation of the University of Zaragoza confirmed that this work was excluded from the scope of application of RD53/2013, of February 1, which established the basic rules applicable for the protection of animals used in experimentation and other scientific purposes, including teaching (36). This committee also confirmed that the design of the study complied with the ethical and animal protection principles used in experimentation and other scientific purposes, including teaching, required by the University of Zaragoza. Ethical approval for the study was granted (PI11/23NE) on 31^st^ March 2023. The cat owners signed informed consent to include their cats in the study and use their data anonymously.

Cats that had previously had an identification microchip implanted were excluded from the study. None of the cats included in this study had a microchip implanted for identification previously and their owners freely decided they would receive a microchip that also provides real–time temperature-sensing.

Three groups of cats were considered: normal, hospitalized and sedated or anesthetized. Normal cats were presented for their first veterinary consultation for vaccination (kittens) or for routine check-ups (adults) and appeared to be healthy. Hospitalized cats were under treatment or observation for at least 1 day for various reasons, including elective surgery (mainly ovariohysterectomy) or clinical conditions (e.g., vomiting, diarrhea, pancreatitis, upper respiratory tract disease, or chronic kidney disease). The group of cats undergoing sedation or anesthesia included both healthy cats requiring chemical restraint to avoid stressful situations and cats that needed surgical or medical treatment. Sedation or anesthesia was carried out in accordance with the protocol in the clinic where the cat was treated.

### Experimental design

There were 58 normal cats (five different breeds and one crossbred cat), 26 hospitalized cats (four breeds) and 36 sedated cats (three breeds). The breed, sex, age and body weight of each cat was recorded. Table 1 shows the characteristics (breed, male frequency, age in months, body weight in kg, and clinic) of the cats included.

**Table 1:** Demographics of cats in the study.

Cats	*n*	Male frequency	Age (months)		Weight (kg)		Breed	Clinic
			Median	IQR	Median	IQR		
Normal	58	32/58 (55.2%)	10.00	20.00	3.30	2.05	Abyssinian: 1/58 (1.7%) Birman: 1/58 (1.7%) European: 52/58 (89.7%) Crossbreed: 1/58 (1.7%) Persian: 2/58 (3.4%) Siamese: 1/58 (1.7%)	A: 1/58 (1.8%); B: 4/58 (6.9%); C:6/58 (10.3%); E:4/58 (6.9%); F: 6/58 (10.3%); G: 6/58 (10.3%); H:7/58 (12.1%); I: 6/58 (10.3%); J:12/58 (20.8%); K: 6/58 (10.3%)
Hospitalized	26	15/26 (57.7%)	13.00	55.00	3.80	2.50	Domestic: 19/26 (73.1%) Long-haired European: 2/26 (7.7%) Maine Coon: 2/26 (7.7%) Persian: 3/26 (11.5%)	B:1/26 (3.8%) G:8/26 (30.8%) H:11/26 (42.3%) J: 6/26 (23.1%)
Sedated/anesthetized	36	17/36 (47.2%)	10.00	8.30	3.35	0.95	Domestic: 34/36 (94.4%) Persian: 1/36 (2.8%); Russian blue: 1/36 (2.8%)	B: 2/36 (5.6%) D: 2/36 (5.6%) E: 2/36 (5.6%) F: 3/36 (8.3%) G: 6/36 (16.7%) H: 7/36 (19.4%) I: 2/36 (5.6%) J: 9/36 (25.0%) K: 3/36 (8.3%)

n, number of individuals; IQR, interquartile range.

### Procedure

A 1.5 mm × 10.7 mm, 0.04 g and 134.20 kHz radiofrequency identification device (microchip) that also provides real–time temperature monitoring (Thermochip® Mini, MSD Animal Health) was implanted subcutaneously at the midpoint of the left side of the neck. The reliability and accuracy (0.07 ± 0.12°C) of the measurements of the temperature-sensing microchip used in the present study have previously been shown at 0 to 40°C in a water bath equipped with an electrical thermometer (37). A pre-packaged sterile positive displacement syringe was used to implant the microchip without sedation. Subcutaneous temperature measurement and animal identification were read simultaneously using a hand-held scanner (SureSense®, MSD Animal Health). No adverse events related to implantation of the temperature-sensing microchip were reported during the study.

In order to minimize the impact of external environmental variables on measurements using the temperature-sensing microchip, the investigator had to ensure that at least 5 min elapsed between the arrival of the cat in the clinic and the first temperature measurement being taken. Since a direct comparison was being made between two methods, this ensured that the environmental conditions under which the temperature measurements were taken were the same for each RT and MT measurement.

Rectal temperature was measured using the COVETRUS digital rectal thermometer with flexible tip VE for veterinary use (Covetrus, Inc). The COVETRUS digital rectal thermometer meets established standards and is ISO 13485 certified. It has a measurement range from 32.0 to 42.9°C, with a precision of ±0.1°C. This rectal thermometer is capable of providing results in just 10 s and issues an audible alarm to notify when the maximum temperature has been reached.

Both MT and RT measurements were carried out by the veterinary team of the participating clinics. In order to avoid factors that could alter the temperature measurement, the investigator had to ensure that the cat was not near to or in contact with sources of heat or cold, such as thermal blankets, water bags, air conditioning or heating system outlets, and stoves. Furthermore, the measurements had to be carried out consecutively to keep the environmental conditions unchanged. All temperature measurements were in degrees Celsius rounded to the nearest single decimal place.

Paired measurements of temperature measurements (MT followed by RT) were taken on three successive occasions (measurements 1, 2, and 3) in each cat. In normal cats, the first and second measurements were taken about 2 weeks apart and the third measurement was about 3 weeks after the second one. In hospitalized cats, the three measurements were taken at three different times on the same day (morning, afternoon, and evening). For cats that were sedated or anesthetized, temperature was measured before, during and after sedation or anesthesia. Each of these paired measurements (MT and RT) was obtained only once on each occasion. To ensure that the handling of the animals was as homogeneous as possible in all collaborating clinics, temperature measurements were taken according to the 2022 AAFP/ISFM Cat Friendly Veterinary Interaction Guidelines (23).

### Statistical analysis

Statistical analysis was carried out with IBM SPSS Statistics 26.0 software (IBM Corp). The level of significance was set at 0.05. Median and interquartile range (IQR) were calculated for both age and body weight, due to the non-normal distribution of these variables, as assessed by the Shapiro–Wilk test for normality. The degree of association between MT and RT was evaluated using both Pearson correlation coefficient *r* and regression modelling (coefficient of determination *R^2^*, coefficients of regression *b*). For the paired measures (MT and RT), a paired t-test was used to assess whether the mean difference (*d*) between the pairs of measurements was zero or not, assuming a normal distribution of differences and with the limits of the 95% confidence interval (*CI*) set at *d* ± 1.96 *SE* (standard error of *d*).

A Bland–Altman plot (difference plot) was used to assess the agreement between the two different measures (MT and RT). The average bias is represented by the gap between the line parallel to x-axis corresponding to no difference and other parallel line corresponding to the mean difference value (*d*). The 95% limits of agreement were calculated using the mean (*d*) and the standard deviation (*SD*) of the differences between the paired measures, assuming normal distribution of differences 95% of differences will be between *d*-1.96 *SD* and *d* + 1.96 *SD*. These limits are represented by dotted lines. Trends in the differences between both temperatures (MT-RT) along the Bland–Altman plot (increasing mean temperature) were investigated using regression analysis. A mixed model approach was used to assess the effects of sex, age and body weight on the differences between MT and RT; these variables were included as fixed effects and the individual was considered as a random effect. For this approach, two age classes (0–12 months; ≥13 months) and two body weight classes (0–3.5 kg and ≥ 3.6 kg) were considered.

## Results

In according to the range of reference intervals for healthy cats said in introduction (18–20), most of RT measurement could be considered as typical of normothermic animals. Of the total RT measurements, only 3.8, 16.7, and 20.0% (normal, hospitalized and sedated/anesthetized cats, respectively) were out these limits.

The relationships and differences between MT and RT, for normal, hospitalized and sedated or anesthetized cats, measurements 1, 2, and 3 and overall (total number of measured pairs) are shown in Table 2. There was a strong, positive, and highly significant relationship between MT and RT in all three groups of cats (*p* < 0.0005). The coefficient of determination *R^2^* can be interpreted as the proportion of the dependent variable (RT) that is predicted by a linear regression model where MT is the independent variable; *R*^2^ranged from 0.5 (measurement 1, normal cats) to 0.9 (most measures in hospitalized and sedated/anesthetized cats). The coefficients of regression *b* were always positive and highly significant (*p* < 0.0005).

**Table 2:** Comparison of MT and RT in cats.

Cats	Parameter	Measure 1	Measure 2	Measure 3	Overall
Normal	*n*	58	54	45	157
	*r* (*p*)	0.7 (<0.0005)	0.8 (<0.0005)	0.8 (<0.0005)	0.8 (<0.0005)
	*R^2^*	0.5	0.7	0.6	0.6
	*b (p)*	0.7 (<0.0005)	0.9 (<0.0005)	1.0 (<0.0005)	0.9 (<0.0005)
	*d ± SD*	−0.1 ± 0.33	−0.1 ± 0.32	0.0 ± 0.28	−0.1 ± 0.31
	*t-*test *p*	0.005	0.054	0.222	<0.0005
Hospitalized	*n*	26	26	26	78
	*r* (*p*)	1.0 (<0.0005)	0.9 (<0.0005)	0.9(<0.0005)	0.9(<0.0005)
	*R^2^*	0.9	0.9	0.9	0.9
	*b (p)*	1.0 (<0.0005)	0.9 (<0.0005)	1.0 (<0.0005)	1.0 (<0.0005)
	*d ± SD*	−0.1 ± 0.19	0.0 ± 0.23	−0.1 ± 0.22	−0.1 ± 0.22
	*t-*test *p*	0.001	0.398	0.125	0.001
Sedated/anesthetized	*n*	34	36	35	105
	*r* (*p*)	0.9 (<0.0005)	0.9 (<0.0005)	0.9 (<0.0005)	0.9 (<0.0005)
	*R^2^*	0.9	0.7	0.9	0.9
	*b(p)*	1.0 (<0.0005)	0.8 (<0.0005)	1.0 (*p* < 0.0005)	1.0 (*p* < 0.0005)
	*d ± SD*	−0.1 ± 0.16	−0.1 ± 0.32	0.0 ± 0.28	−0.1 ± 0.26
	*t-*test *P*	<0.0005	0.031	0.468	0.001

MT, microchip temperature; RT, rectal temperature; n, MT and RT paired measures; r, correlation coefficient for MT and RT paired measures; *R*^2^, coefficient of determination for MT and RT; b, coefficient of regression for MT and RT; d, Mean difference for MT and RT; SD, standard deviation; P, significance.

Mean differences between MT and RT were always negative. These mean differences were significantly different from 0 (*p* < 0.05) for measurement 1 and overall, in both normal and hospitalized cats, and for measurements 1, 2 and overall, in sedated cats. In normal cats, the overall average bias of MT compared to RT was −0.1°C (*p* < 0.0005; limits of 95% *CI*: −0.14°C and − 0.04°C). In hospitalized cats, the average bias was −0.1°C (*p* = 0.001; limits of 95% *CI*: −0.13°C and −0.04°C). The average bias in sedated or anesthetized cats was −0.1°C (*p* = 0.001; limits of 95% *CI*: −0.14°C and −0.04°C).

**Figure 1:**
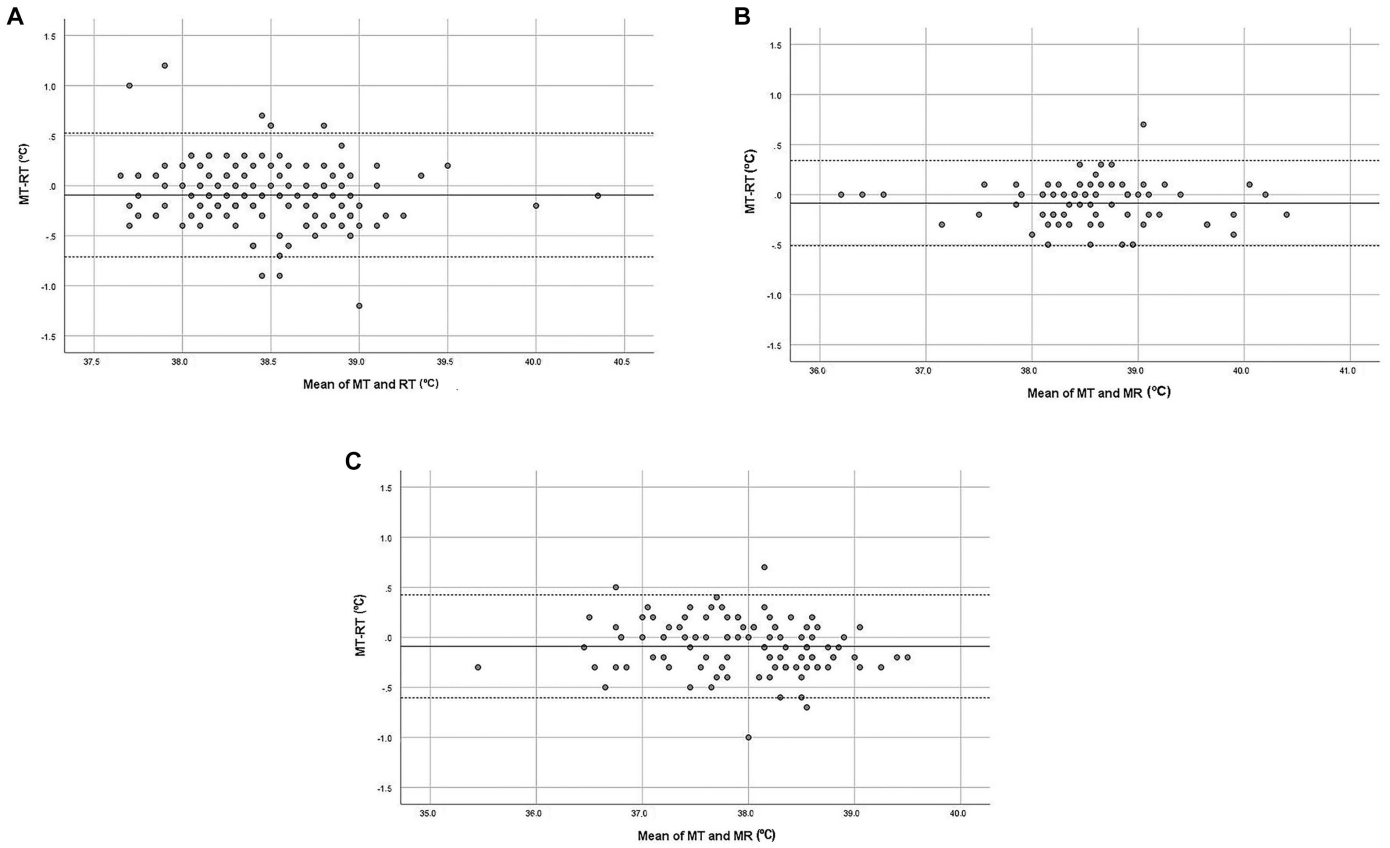
Bland–Altman plot demonstrating the relationship between MT (microchip temperature, °C) and RT (rectal temperature, °C) in cats (overall data). **(A)** Normal cats, **(B)** Hospitalized cats, **(C)** Sedated/anesthetized cats. The *x*-axis represents the mean of MT and RT (°C) for each pair of data. The *y*-axis represents the difference between MT and RT for each pair of data. The bias is represented by the gap between the *x*-axis, corresponding to zero differences, and the parallel continuous line to the *x*-axis at *d* (mean of differences between MT and RT). Dotted lines represent the 95% limits of agreement.

Figure 1 shows the Bland–Altman plots for all measurements (overall) from normal, hospitalized, and sedated cats (A, B, and C, respectively).

In normal cats, the 95% limits of agreement were − 0.71°C and 0.53°C (Figure 1A). A negative trend was evidenced along the graphic (*F* = 4.794; *p* = 0.030): the differences between MT and RT decreased as the mean temperature increased (*b* = −0.1). There was no effect of sex (*F* = 2.531; *p* = 0.117), age (*F* = 1.514; *p* = 0.224) or body weight (*F* = 0.715; *p* = 0.401) on the differences between MT and RT. The 95% limits of agreement were −0.51 and 0.34°C for hospitalized cats (Figure 1B). Proportionality between the difference and the mean of the measurements was not detected (*F* = 0.107; *p* = 0.745). There was no effect of sex (*F* = 0.915; *p* = 0.349), age (*F* = 0.026; *p* = 0.874), or body weight (*F* = 2.676; *p* = 0.116) on the differences. For sedated or anesthetized cats, the 95% limits of agreement were −0.60 and 0.42°C (Figure 1C). As for hospitalized cats, there was no significant trend (*F* = 2.962; *p* = 0.088) and no effect of sex (*F* = 0.824; *p* = 0.371), age (*F* = 0.484; *p* = 0.492) or body weight (*F* = 0.006; *p* = 0.940).

## Discussion

The study examined the use of a temperature-sensing microchip in normal, cats, hospitalized cats and sedated/anesthetized, aiming to assess its effectiveness in measuring body temperature. It is important to acknowledge certain limitations within this study. These limitations primarily arise from the low number of MT measurements due to the absence of a mandatory cat identification requirement in Spain until recently. To address these limitations, future research should focus on increasing the sample size to obtain more robust and representative data. The main limitation of this study was that the accuracy and reliability of the subcutaneously implanted temperature-sensing microchips was mostly performed in animals that were normothermic. In order to fully assess the clinical usefulness of these devices in cats further research is required to assess the accuracy and reliability when they have abnormal body temperatures, especially during febrile conditions, but also when they develop hyperthermia from other causes, and when they are hypothermic.

Additionally, it is crucial to note that identification microchips are implanted subcutaneously, thus the measurement of MT (microchip temperature) was taken in close proximity to the skin surface of the cats, which may introduce variability in the results. Moreover, the generalizability of the findings may be limited by the fact that the performance of the temperature-sensing microchip was not evaluated under different environmental conditions. Efforts were made to minimize potential environmental influences on temperature readings. Collaborating clinics were provided with specific instructions on how to avoid external factors that could affect the measurements, such as contact with heat pads, hot water bottles, air conditioning, or other heat sources. Cats were also given an acclimatization period in a controlled clinic environment before the temperature measurements were taken, reducing the impact of outdoor conditions. To minimize stress in the cats, only a single paired measurement of MT and rectal temperature (RT) was obtained on each occasion. This approach was chosen to avoid subjecting the cats to multiple consecutive rectal measurements, which could potentially affect their stress levels and subsequently body temperature. While these limitations should be considered, the authors took necessary precautions to maintain the reliability and validity of the study findings. Further research with a larger sample size and evaluation of the performance of the temperature-sensing microchip performance under various environmental conditions would provide valuable insights.

Subcutaneously implanted temperature-sensing microchips may not always provide a reliable measurement of core body temperature, depending on ambient environmental conditions in animals housed outdoors. Temperature-sensing microchips were recognized as a practical method for estimating body temperature in beef cattle on pasture, but were less precise than RT measurement (38). Moreover, core temperature changes caused by the cold challenge did not correlate well with temperature-sensing microchip measurements (39). The site of temperature-sensing microchip implantation effects the closeness of agreement between and core and rectal temperature measurements in goats (40). Temperature-sensing microchips provided an approximate correlate of body temperature in groups of pigs, but were not a suitable tool for the measurement of temperature in individual animals in an infection model (41). Correlation and regression analysis are two statistical techniques that have been used frequently to study the relationship between two methods of temperature measurement (27, 42–45). Correlation analysis, which helps to measure the magnitude and direction of the degree of association between two variables (positive or negative correlation), is typically favored for clinical applications (42). Regression analysis provides a mathematical equation that describes the directional relationship (positive or negative) between two (or more) variables that can be used to predict whether the value of one variable can be based on the value of the other. In other words, both statistical methods evaluate the relationship between two sets of variables but not their agreement (46); where the correlation coefficient *r* is a measure of the strength of the relationship between the two variables and the coefficient of determination *R^2^* represents the proportion of variance in the dependent variable (temperature measurement) that can be explained by the independent variable (measurement method) (47). Given that two methods for measuring the same variable (temperature) are inherently related, testing for statistical significance usually confirms this (48). Therefore, the significant and high values obtained for both *r* (correlation coefficient) and *R^2^* (coefficient of determination) in the present study are not unexpected. This means that the significant and high values for both *r* and *R^2^* obtained in the present work are not unexpected. In ferrets, the correlation between MT and RT (digital thermometer) was good; MT showed sufficient agreement with RT, providing a reliable alternative for measuring temperature (44). Similarly, our *r* values were always high and positive. However, it is important to note that the strength and direction of correlation will depend on various factors, such as the time of day, body position, and the health of the individual.

To assess the comparability between the two methods, differences must be highlighted (48). Thus, Altman and Bland proposed an alternative analysis for the quantification of the agreement between two quantitative measurements, based on the mean difference *d* and the subsequent construction of limits of agreement (49, 50). The effectiveness of temperature-sensing microchips as a useful alternative method for measuring temperature was demonstrated using the mean differences between MT and RT in common marmoset (0.26°C ± 0.02; 11) and rat and mouse (not significantly different from zero; 32). There were significant differences between RT and MT in horses and sheep, but not in goats (33). Close agreement between MT and RT has been reported for cats (51). Another study reported that MT yielded slightly lower temperatures readings than RT in pigs (52).

As stated previously, the reliability and accuracy of the measurements of the temperature-sensing microchip used in the present study was 0.07 ± 0.12°C. In our study, the mean differences between MT and RT (average biases) were always negative and there were no significant mean differences (*d*) for measurements 2 and 3 in the three groups of cats. Small sample size is a possible explanation for the lack of significant results. The overall mean differences (*d*) were all negative and significantly different from zero. A negative and statistically significant *d* value means that MT is usually lower than RT. As shown in Table 2, the overall *d* values were −0.1. In addition, every significant *d* value was below 0.5°C, an arbitrary cut-off point that has been assumed to be an acceptable error in temperature readings in clinical settings in dogs and cats (29, 53–55). This means that, although statistically significant, these *d* values could be considered as biologically unimportant.

The mean difference *d* is an average estimation of bias, but individual data are important because individuals may respond in unique ways (27), even if animals with the same status (normal, hospitalized, sedated/anesthetized) are considered. The 95% limits of agreement should contain the difference between MT and RT for 95% of individual paired measures and therefore, enable better understanding of the differences for every pair of measurements. As shown in Figure 1, the dots representing the differences between MT and RT are spread evenly between the 95% limits of agreement, with only a few points outside of these limits, meaning that the bias could be positive or negative, depending on the temperature measurement. Thus MT, while close to RT, is not identical to it. It would be interesting to assess MT and RT temperature measurements over time under different conditions and compare these. Temperature measurements from different sites (such as RT and ear canal temperature) are related, tend to follow similar patterns under different conditions (such as at rest and after exercise) but are not identical (56). Thus, it may be possible that variations in MT could potentially serve as an indicator for predicting variations in RT but would have to be confirmed by frequent RT measurement, which is challenging. However, caution must be taken when interpreting a single measurement of subcutaneous temperature using a temperature-sensing microchip as it may be influenced more by external temperature factors (e.g., a heat blanket) than RT measurements.

Although the aim of this work was not compare temperature in cats under different conditions (normal, hospitalized, sedated/anesthetized), it is clear that differences in temperature between individuals are connected to their health status. Hyperthermia can be produced in rats using neurotoxic agents, which affect central thermoregulation (57). Also, nociceptive stimuli can cause temperature changes, due to activation of the sympathetic adrenomedullary system [released catecholamines activate adrenoceptors in vascular smooth muscle, which alters blood flow, blood pressure, heart rate and respiratory rate and can alter both peripheral and core temperature (58)]. The Bland–Altman method alone cannot determine whether the 95% limits of agreement set are acceptable or not. Therefore, clinical or biological considerations must be used to define acceptable limits (47). Limits for maximum acceptable differences should be determined *a priori* and may depend on the species and method of temperature measurement. In human studies, core temperature and RT measurements had an acceptable level of agreement of 0.1°C, with 95% limits of agreement within 0.3°C (59) or 0.4°C (60). In veterinary literature, acceptable limits of agreement and degree of bias have not been universally agreed upon. For laboratory rabbits, acceptable limits of agreement for MT and RT were less than 2°C (61). In goats, temperature-sensing microchips implanted retroperitoneally had the closest agreement (mean 0.2°C lower) with RT (40). For dogs, around 0.5°C was considered an acceptable limit of agreement, as temperature differences greater than this could impact diagnostic and treatment decisions (54). Expert opinion based on a small sample of dogs (*n* = 16) also suggests that a maximal clinical tolerance of 0.5°C is acceptable (53). For cats, the predetermined cut-off for the calculated limits of agreement between RT and MT has been set at 0.83°C, based on acceptable differences between two methods of temperature measurement used in a FHV1 (feline herpesvirus 1) study (51). Our 95% limits of agreement are within the 0.83°C proposed by Quimby et al. (51). Therefore, the agreement between MT and RT in our study appears to be acceptable. Significant trends in the difference between MT and RT mean that the differences are proportional to the magnitude of the temperature measurement. A significant negative trend was only found for normal cats; however, the *b* value was low, pointing to only a small decrease in the difference between MT and RT as the value of the measured temperatures increases. There was no effect of age and body weight.

## Conclusion

Body temperature is an essential health parameter in cats that helps inform the clinical decisions made by practicing veterinarians. RT measurement is difficult or may even be impossible to perform and can lead to a high degree of stress. The use of temperature-sensing microchips may provide a good alternative for the measurement of body temperature in cats. In veterinary practices, microchip temperature may provide an easy and time-saving way to assess temperature variations for hospitalized animals. Temperature-sensing microchips allow both identification of the individual and assessment of body temperature. This type of microchip should make measuring temperature easy, fast, safe, and less stressful for both the cat and the veterinary team. This study pointed to that MT would be expected to be slightly below RT in cats. The average bias (*d*) was always negative, and these *d* values are considered not to be important biologically. In addition, the 95% limits of agreement were narrow enough to satisfy the acceptability conditions established previously for cats (0.83°C). However, the accuracy and reliability of the subcutaneously implanted temperature-sensing microchips was mostly performed in animals that were normothermic. Therefore, further studies in larger groups of cats under different conditions are needed to compare trends and assess variation with time.

## Data availability statement

The raw data supporting the conclusions of this article will be made available by the authors, without undue reservation.

## Ethics statement

The animal studies were approved by The Ethical Advisory Committee for Animal Experimentation of the University of Zaragoza. The studies were conducted in accordance with the local legislation and institutional requirements. Written informed consent was obtained from the owners for the participation of their animals in this study.

## Author contributions

MG: Conceptualization, Data curation, Formal analysis, Funding acquisition, Investigation, Methodology, Project administration, Resources, Supervision, Validation, Visualization, Writing – original draft, Writing – review & editing. JG: Conceptualization, Data curation, Formal analysis, Funding acquisition, Investigation, Methodology, Project administration, Resources, Supervision, Validation, Visualization, Writing – original draft, Writing – review & editing. MT: Conceptualization, Data curation, Formal analysis, Methodology, Software, Writing – original draft, Writing – review & editing. FB: Conceptualization, Data curation, Formal analysis, Funding acquisition, Investigation, Methodology, Project administration, Resources, Supervision, Validation, Visualization, Writing – original draft, Writing – review & editing.
